# What is the preferred mesh placement in primary ventral hernia repair? An international survey of 442 surgeons

**DOI:** 10.1007/s10029-025-03429-1

**Published:** 2025-09-09

**Authors:** Usamah Ahmed, Jacob Rosenberg, Sarfaraz Jalil Baig, Sujith Wijerathne, Wah Yang, Shuqing Li, Jason Joe Baker

**Affiliations:** 1 Center for Perioperative Optimization, Department of Surgery, Copenhagen University Hospital - Herlev and Gentofte, Borgmester Ib Juuls Vej 1, Herlev, DK-2730 Denmark; 2 Digestive Surgery Clinic, 7B, St Francis Xavier Sarani, Mullick Bazar, Park Street area, Kolkata, 700017 West Bengal India; 3 Department of Surgery, Alexandra Hospital, 378 Alexandra Road, 159964 Singapore, Singapore; 4 Department of Metabolic and Bariatric Surgery, The First Affiliated Hospital of Jinan University, 510632 Guangzhou, China; 5 School of Clinical Medicine, Jiangxi University of Chinese Medicine, Mei Ling Da Dao No.1688, Nanchang, 330004 Jiangxi China

**Keywords:** Ventral hernia, Chronic pain, Recurrence, Foreign body sensation, Retromuscular, Preperitoneal

## Abstract

**Purpose:**

Primary ventral hernia repair is a common elective procedure; however, mesh placement practices vary widely, and there is limited evidence to guide optimal placement. This international study examined surgeons’ preferences and considerations regarding mesh placement in elective primary ventral hernia repair.

**Methods:**

We conducted an international cross-sectional survey targeting surgeons experienced in primary ventral hernia repair. The survey was distributed through hernia societies and social media platforms. It included 31 questions addressing surgeon demographics and their beliefs on various mesh placements. Data were collected using REDCap, Google Forms, and Questionstar.

**Results:**

A total of 442 surgeons participated, with the majority being specialist surgeons (96%) who had performed at least 100 repairs (82%). Inlay was the least familiar mesh technique (26%). For hernia defects < 1 cm, preperitoneal (28%) and suture-only repair (27%) were considered to yield the best overall outcomes. For defects ≥ 1 to ≤ 4 cm, preperitoneal and retromuscular techniques were equally favored (34%), whereas retromuscular was regarded as the best option for larger defects (> 4 to 9 cm; 68%). Laparoscopic and robotic-assisted approaches were increasingly preferred for larger defect sizes. Hernia defect size (93%), surgical history (90%), and obesity (80%) were the most common factors influencing the choice of mesh placement.

**Conclusion:**

Preperitoneal and suture-only repairs were most commonly selected for hernia defects < 1 cm, while preperitoneal and retromuscular placements were equally favored for defects ≥ 1 to ≤ 4 cm. For defects > 4 to 9 cm, retromuscular placement was selected by most surgeons. As defect width increased, laparoscopic and robot-assisted approaches gained preference. Key factors influencing decisions included hernia defect size, surgical history, and obesity. The lack of strong supporting evidence highlights the need for further high-quality research.

## Introduction

The practice of primary ventral hernia repair varies not only internationally but also across different centers within the same country [1–4]. It is a common surgical procedure that can be performed with or without the use of mesh [5–7]. Mesh may be placed by open, laparoscopic, or robot-assisted approaches, but with variations of which anatomical layers it is positioned [7–9]. Accessing and placing mesh in different anatomical planes can lead to varying complications, including pain, foreign body sensation, and impacts on overall quality of life [10, 11]. These issues may arise from fascial dissection or a reaction to the mesh itself [10]. A range of factors might guide surgeons’ decisions regarding mesh placement, such as available published evidence, personal experience with specific cases, and patient characteristics such as sex, age, and medical and surgical history. Additionally, the nature of the hernia, whether the case is elective or emergency, along with its size and location, can play a critical role [12]. Organizational factors, such as the level of local expertise, available resources, and cost considerations, also impact these decisions, as intraperitoneal onlay mesh (IPOM) placement often requires more costly meshes due to their compositions, such as anti-adhesive coatings [13]. Furthermore, current guidelines suggest preperitoneal or retromuscular placement albeit based on low quality evidence [14].

Previous surveys have explored surgeons’ perspectives on indications, contraindications, and techniques for ventral hernia repair [1, 2, 4, 15–17]. However, most of these studies focused on complex or incisional hernias or did not specifically address mesh placement and patient-reported outcomes. As a result, there remains a gap in understanding surgeons’ considerations regarding mesh placement in primary ventral hernia repair. To our knowledge, no studies have examined the specific factors surgeons consider, such as defect sizes, when selecting the anatomical plane for mesh placement in primary ventral hernia repairs.

Therefore, this study investigated surgeon preferences and considerations when selecting anatomical layers for mesh placement in elective primary ventral hernia repair. A secondary objective was to identify patient characteristics influencing the selection of mesh location.

## Methods

### Study design and setting

This study is reported in accordance with the Strengthening the Reporting of Observational Studies in Epidemiology (STROBE) guidelines and the Checklist for Reporting Of Survey Studies (CROSS) guidelines [18, 19]. This cross-sectional study utilized a web-based open survey, administered through Research Electronic Data Capture (REDCap) [20], Google Forms [21], and Questionstar (survey tool in China) [22]. The survey was distributed internationally, and responses were collected between September 24, 2024, and March 1, 2025.

### Participants

The inclusion criteria consisted of surgeons from around the world who had experience in performing elective primary ventral hernia repairs. A time-constrained convenience sampling method was employed to recruit participants.

### Data collection methods

The study was conducted using a self-developed survey consisting of seven sections and contained a total of 31 items, all of which were mandatory [23]. The first section included an introduction page outlining the aim of the survey and specifying that it was intended for surgeons. The second section collected information on surgeon demographics, while the third section focused on the types of hernia repairs the surgeons had experience performing. The fourth section focused on patient characteristics that influence the surgeon’s choice of mesh placement. A comment box was provided to offer additional remarks on patient characteristics if necessary. In the fifth section, surgeons were asked about their preferred surgical approach. The sixth section explored surgeons’ perspectives on mesh placement and its associated complications. Finally, the seventh section asked surgeons to identify the mesh placement they believed would achieve the best overall outcome. From section five onward, questions were stratified by hernia defect width, categorized as < 1 cm, ≥ 1 to ≤ 4 cm, and > 4 to 9 cm. To ensure consistent terminology, an illustration of the anatomical planes was included in the survey (adapted from [24]). Mesh placements were defined as follows: onlay if positioned superficially to the anterior rectus fascia; retromuscular if placed between the rectus muscle and the posterior rectus fascia; preperitoneal if placed between the posterior rectus fascia and the parietal peritoneum; and IPOM if positioned on the visceral side of the parietal peritoneum. Inlay placement was defined as mesh positioned within the fascial defect and fixated to the edges of the hernia defect. Participants were able to review and modify their responses before submission.

### Study preparation and administration

During the development of the survey, it underwent initial face validation with two medical doctors. They completed the survey under observation and were asked to explain each question after reading it. Any parts of the survey that were not interpreted as intended were revised and refined after each round of validation. The survey was then pilot-tested with three hernia experts and further adapted based on their feedback. The survey was distributed from September 24, 2024, to March 1, 2025, spanning a period of just over five months. Participation was voluntary, and informed consent was obtained through participants’ active opt-in at the beginning of the survey. The survey’s purpose was clearly stated on the introduction page, and no personal or identifying information was collected to ensure participant anonymity. The survey was open in design, meaning it was accessible to all visitors of the survey site. To reduce the risk of unintended participation, the introduction page explicitly stated that the survey was intended for surgeons with experience in performing primary ventral hernia repairs. Surgeons who indicated that they had not performed any primary ventral hernia repairs were automatically excluded from completing the rest of the survey, as their input was considered not meaningful to the study.

The survey link was primarily distributed via the mailing lists of the Danish Hernia Database, the Abdominal Core Health Quality Collaborative (ACHQC), the Hernia Society of India (HSI), and the Asia-Pacific Hernia Society (APHS), as well as through newsletters of the European Hernia Society (EHS). In addition, the survey was also shared via WhatsApp groups for HSI and APHS. The survey was further posted on the official website of the Danish Hernia Database. In addition, the survey was promoted on social media platforms by the Danish Hernia Database, EHS, and the Canadian Hernia Society, as well as by influential figures in the hernia surgery community. It was also shared in professional Facebook groups, including the International Hernia Collaboration (IHC) and the Abdominal Wall Reconstruction (AWR) Surgeons Community. In China, distribution was carried out through regional surgical networks and local professional groups. To enhance participation, the survey was periodically reposted and redistributed through the mailing lists during the data collection period. Participants accessed the survey through links to REDCap (primarily in Denmark), Questionstar (in China), or Google Forms (for the rest of the world). Participants were instructed to participate only once using one of the provided links. Incomplete surveys were included in the analysis, with no imputation performed for missing data.

### Statistical analysis

Data from REDCap, Questionstar, and Google Forms were merged for analysis. Categorical variables were reported as counts and percentages. Continuous variables were assessed for normality using histograms and the Shapiro-Wilk test. As all continuous data were non-normally distributed, they were presented as medians with interquartile ranges. Statistical analyses were performed using IBM SPSS Statistics, version 29 (IBM Corp., Armonk, NY, USA). A world map depicting the countries of residence of participating surgeons was generated using Microsoft Excel.

### Ethical considerations

No formal approval for data collection and storage was required, as the survey was anonymous, and no personal data were collected. According to Danish legislation, this type of study does not require approval from an ethics committee.

## Results

**Fig. 1:**
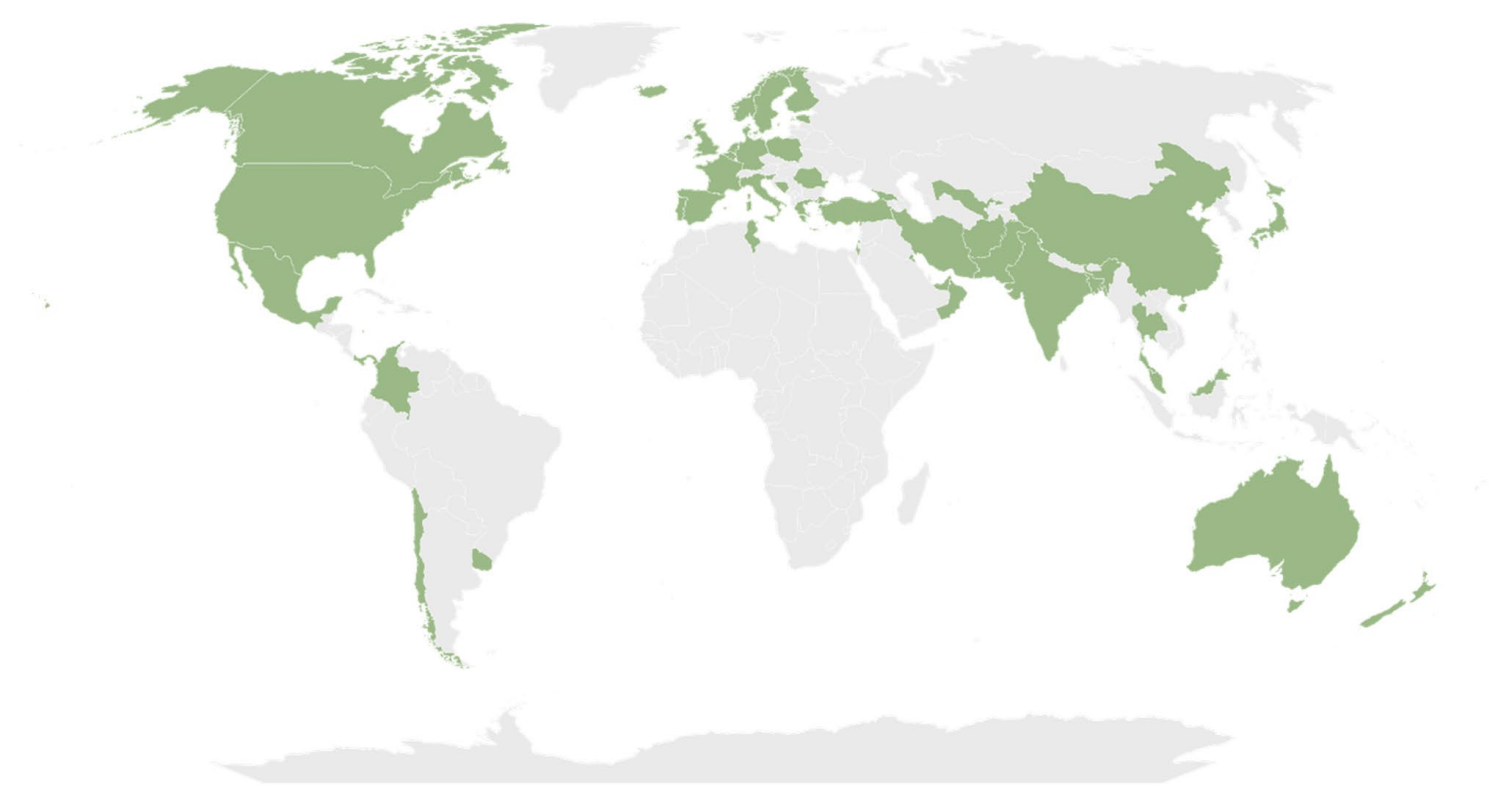
World map showing the countries of participating surgeons. Shaded areas represent countries from which survey responses were received

A total of 442 responses were recorded, of which 21 were incomplete (5%) (Table 1). Approximately one in eight respondents were female, and the median age of participating surgeons was 48 years. Nearly all respondents were specialist surgeons (96%), with more than half having graduated as medical doctors over 20 years ago. Most participants resided in Asia (*n* = 264, 60%), followed by Europe (*n* = 114, 26%), North America (*n* = 56, 13%), and other regions (*n* = 8, 2%) (Fig. 1). Four out of five surgeons reported having performed at least 100 primary ventral hernia repairs, and nearly half had completed more than 500 repairs. Most surgeons had experience with all mesh placement techniques except for inlay repair, which was the technique with the least reported experience. When considering patient characteristics that influence mesh placement decisions, hernia defect size was most frequently selected (*n* = 412, 93%), followed by surgical history (*n* = 399, 90%) and obesity (*n* = 352, 80%). Other factors included age (*n* = 224, 51%), medication use (*n* = 179, 41%), diabetes (*n* = 175, 40%), smoking (*n* = 153, 35%), sex (*n* = 95, 22%), and alcohol misuse (*n* = 76, 17%). Additionally, through open comments, some surgeons mentioned inflammatory bowel disease (IBD), cost considerations, rectus diastasis, and cardiopulmonary diseases as factors influencing their decisions.

**Table 1:** Surgeon demographics

Demographics	
Responses	442
Incomplete	21 (5)
Female sex	53 (12)
Age, median [IQR]	48 [40–55]
Specialist surgeon	423 (96)
Years since graduation as MD	
< 10	44 (10)
10–20	155 (35)
21–30	140 (32)
> 30	103 (23)
N repairs performed in total	
1–49	34 (8)
50–99	47 (11)
100–500	164 (37)
> 500	197 (45)
Types of repairs performed	
Onlay	334 (76)
Inlay	115 (26)
Retromuscular	376 (85)
Preperitoneal	348 (79)
Intraperitoneal (IPOM)	373 (84)
Suture (no mesh)	315 (71)

Values are n (%) unless otherwise indicated. n, number of surgeons; N, number; IQR, interquartile range; MD, medical doctor; IPOM, intraperitoneal onlay mesh

Regarding surgical approach, robot-assisted procedures were increasingly preferred as hernia defect width increased (Table 2). For defects < 1 cm, most surgeons preferred an open approach, followed by laparoscopic approach. For defects ≥ 1 to ≤ 4 cm, laparoscopic repair was the preferred method. In cases of larger defects > 4 to 9 cm, laparoscopic, open, and robotic approaches were preferred with a more balanced distribution.

**Table 2:** Preferred surgical approach, stratified by hernia defect width. Surgeons were allowed to select only one surgical approach per size category

	< 1 cm	≥ 1 to ≤ 4 cm	> 4 to 9 cm
Open	290 (66)	87 (20)	150 (34)
Laparoscopic	141 (32)	284 (64)	183 (41)
Robot-assisted	11 (3)	71 (16)	109 (25)

Values are n (%). Percentages in parentheses represent the proportion of total participants (*n* = 442). n, number of surgeons

**Fig. 2:**
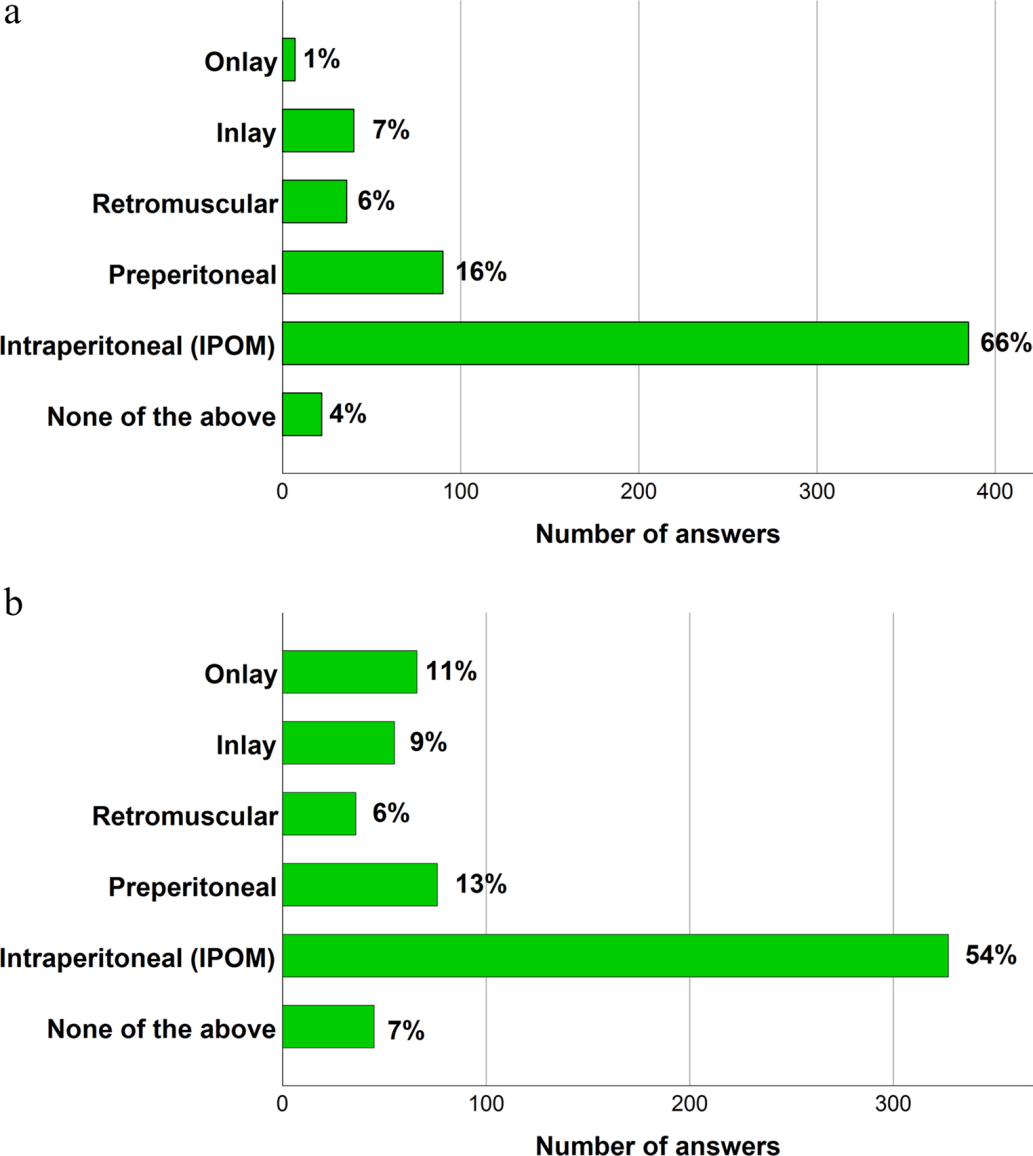
Mesh placements surgeons believe to pose a clinical concern for bowel adhesion and obstruction (2**a**), and fistula formation (2**b**). IPOM, intraperitoneal onlay mesh

Surgeons’ beliefs of the mesh placement least likely to result in complications varied by hernia defect width (Table 3). For defects < 1 cm, the surgeons most frequently believed that preperitoneal placement carried the lowest overall risk (35–45%), followed by retromuscular (17–29%) and IPOM (12–27%). However, for recurrence and chronic pain specifically, onlay was believed by many surgeons to be more favorable than both retromuscular and IPOM. For defects ≥ 1 to ≤ 4 cm, retromuscular placement was most commonly believed to be the safest option (36–42%), followed by preperitoneal (29–40%) and IPOM (13–24%). In larger hernia defects (> 4 to 9 cm), retromuscular placement was clearly favored, with 50–75% of surgeons believing the mesh plane to be associated with the lowest complication risk, while preperitoneal was selected by 11–28% of surgeons. Across all hernia defect widths and complication categories, inlay placement was consistently considered the least favorable, with very few surgeons believing it to carry the lowest risk. Two out of three surgeons considered IPOM as a clinical concern for bowel adhesion and obstruction (Fig. 2a), while preperitoneal placement was also considered to carry a risk, though by much fewer surgeons. Additionally, more than half of surgeons considered IPOM a clinical concern for fistula formation (Fig. 2b). Surgeons’ beliefs of which repair technique would yield the best overall outcome also varied by hernia defect width (Table 4). For defects < 1 cm, the most commonly selected placements were preperitoneal repair and suture-only repair without mesh, followed by onlay repair. For defects ≥ 1 to ≤ 4 cm, preperitoneal and retromuscular repairs were equally believed to have the best outcome, followed by IPOM. For larger defects measuring > 4 to 9 cm, retromuscular repair was overwhelmingly favored, with 68% of surgeons believing this placement to be the best.

**Table 3:** Mesh placement surgeons believed to carry the lowest risk of postoperative complications, stratified by hernia defect width. Surgeons were allowed to select only one mesh placement per complication category

	Onlay	Inlay	Retromuscular	Preperitoneal	Intraperitoneal (IPOM)
Defect width < 1 cm					
Recurrence	96 (22)	13 (3)	74 (17)	163 (37)	86 (20)
Chronic pain	87 (20)	15 (3)	77 (17)	194 (44)	55 (12)
Foreign body sensation	38 (9)	16 (4)	107 (24)	198 (45)	69 (16)
Surgical site infection	30 (7)	16 (4)	127 (29)	166 (38)	85 (19)
Seroma	37 (8)	19 (4)	94 (21)	155 (35)	117 (27)
Defect width ≥ 1 to ≤ 4 cm					
Recurrence	18 (4)	11 (3)	172 (39)	128 (29)	105 (24)
Chronic pain	24 (5)	8 (2)	162 (37)	177 (40)	58 (13)
Foreign body sensation	11 (3)	11 (3)	173 (39)	163 (40)	70 (16)
Surgical site infection	12 (3)	12 (3)	187 (42)	133 (30)	80 (18)
Seroma	16 (4)	9 (2)	157 (36)	132 (30)	108 (24)
Defect width > 4 to 9 cm					
Recurrence	17 (4)	2 (1)	330 (75)	48 (11)	37 (8)
Chronic pain	20 (5)	3 (1)	263 (60)	115 (26)	28 (6)
Foreign body sensation	11 (3)	5 (1)	245 (55)	112 (28)	45 (10)
Surgical site infection	9 (2)	9 (2)	257 (58)	89 (20)	60 (14)
Seroma	14 (3)	5 (1)	222 (50)	103 (23)	78 (18)

Values are n (%). Percentages in parentheses represent the proportion of total participants (*n* = 442). n, number of surgeons; IPOM, intraperitoneal onlay mesh

**Table 4:** Repair technique believed to provide the best overall outcome, stratified by hernia defect width. Surgeons were allowed to select only one repair technique per size category

	< 1 cm	≥ 1 to ≤ 4 cm	> 4 to 9 cm
Onlay	74 (17)	17 (4)	12 (3)
Inlay	13 (3)	7 (2)	4 (1)
Retromuscular	47 (11)	150 (34)	301 (68)
Preperitoneal	122 (28)	149 (34)	72 (16)
Intraperitoneal (IPOM)	48 (11)	93 (21)	30 (7)
Suture (no mesh)	117 (27)	5 (1)	2 (1)

Values are n (%). Percentages in parentheses represent the proportion of total participants (*n* = 442). n, number of surgeons; IPOM, intraperitoneal onlay mesh

## Discussion

Our findings suggest that the repair technique surgeons considered to yield the best overall outcome varied according to the width of the hernia defect. For smaller hernia defects, the suture-only and preperitoneal mesh placements where mainly believed to be the best technique. However, as the defect size increased, retromuscular mesh placement became increasingly favored, with over two-thirds of surgeons selecting it for defects > 4 cm. Additionally, laparoscopic and robot-assisted approaches were more commonly preferred as hernia defect width increased.

Our observations contrast with the results of an online case-based survey from 2017, which was conducted in the Facebook group International Hernia Collaboration (IHC). The case-based survey presented a specific patient case with a hernia defect of 5 × 6 cm. In that survey, most surgeons answered that they favored the laparoscopic IPOM approach [2]. However, several factors limit the comparability between the two studies. The case-based survey focused on one patient with an incisional hernia and a certain defect size, whereas our survey addressed primary ventral hernias in general and not exclusively one case, and there were thus three conditions that differed in risk profiles and may warrant different treatment strategies [25]. Additionally, there was a seven-year gap between the surveys, during which new guidelines for mesh placement were published [14, 26]. At the time of the case-based survey, available guidelines did not provide specific recommendations on mesh positioning [27]. Another case-based survey from 2021 reported that IPOM, retromuscular, and preperitoneal approaches were used with similar frequency among surgeons [17]. This survey also focused on one patient case with incisional hernia and only included surgeons from Canada, limiting the generalizability of its findings to a global context. In contrast, our survey provides international insights into surgeons’ current preferences, which, for most surgeons, appear to align with current recommendations from guidelines, particularly for larger hernia defects [14, 28]. In hernia defects ≤ 4 cm, however, surgeon preferences were more varied. This reflects an ongoing debate on the optimal mesh placement for elective primary ventral hernia repair, as the current guidelines on mesh placement are based on very low certainty of evidence [14, 28, 29]. Our findings suggest a shift away from IPOM as a precautionary measure to reduce the risk of bowel adhesions and obstruction, based primarily on case reports and small series [14, 30, 31]. This is reflected in surgeons’ selection of the best overall mesh placement, which indicates a trend away from IPOM compared with previous survey studies [2, 17]. Although some observational studies have examined the risks of bowel adhesions and obstruction, they often lack comparators or sufficient power to draw definitive conclusions regarding the safety of IPOM [32, 33]. As such, current trends may be shaped more by concern over complications than by strong evidence. This highlights the need for high-quality, well-powered trials so that surgical decision-making can be guided by robust data rather than prevailing practice or local preference.

In addition to hernia defect size, our findings show that surgeons also considered previous surgical history and obesity as important factors influencing the selection of mesh placement. Prior surgical history may lead to intraabdominal adhesions, which can complicate laparoscopic procedures and influence the feasibility of certain mesh planes [34]. These considerations are particularly relevant when selecting the optimal mesh placement, as they may directly impact surgical outcomes. In our study, laparoscopic approaches were commonly preferred for hernias ≥ 1 cm, potentially reflecting efforts to reduce infection risk [35]. Additionally, multiple studies have shown that obesity and smoking are associated with an increased risk of wound complications and infections following mesh repair [36, 37]. However, to our knowledge, little to no evidence exists on mesh placement depending on patient characteristics such as obesity and surgical history. Given these limitations, further research is needed to strengthen the evidence base and guide surgeons toward the safest and most effective mesh placement for patients.

### Strengths and limitations

The strength of this study is that it includes a high number of responses from 442 surgeons worldwide. To our knowledge, this is the first international survey to explore surgeons’ perspectives and considerations regarding mesh placement in primary ventral hernia repair. Conducted with a cross-sectional design, the survey provides a snapshot of current preferences and decision-making among hernia surgeons internationally. The high level of experience among respondents in performing hernia repairs and using various mesh placement techniques supports the validity of the insights gathered. The study further demonstrates that surgeons’ beliefs largely align with existing guidelines despite the lack of high-quality evidence supporting specific approaches. The survey instrument was developed in collaboration with hernia experts and underwent both face validation and pilot testing, enhancing its relevance and clarity.

However, several limitations should be acknowledged. Although the survey received 442 responses, the majority originated from Asia, with additional representation from Europe and North America. Responses from South America, Oceania, and Africa were close to absent, limiting the generalizability of the findings to these regions. Geographic bias may also have been introduced due to the inability to distribute the survey in certain countries, such as Russia and North Korea, where access was restricted either by the survey tool providers or by local regulations. This may have skewed the data toward Western and Asian surgical practices. Furthermore, due to the open design of the survey and its distribution via both social media and mailing lists, we were unable to determine the total number of recipients or views, and therefore could not calculate a response rate. This limits our ability to assess the overall level of interest among the surgeons. Additionally, the use of social media platforms may have resulted in overrepresentation of surgeons who are active in such digital spaces, potentially reinforcing dominant professional perspectives while underrepresenting those less engaged in these forums.

### Perspectives

Beliefs for mesh placement providing the best outcome in elective primary ventral hernia repair have shifted in recent years toward retromuscular and preperitoneal placements [31]. This shift may be influenced by newly developed guidelines that address mesh placement more explicitly, although these guidelines are still constrained by low-certainty evidence. The move away from IPOM appears to be driven primarily by concerns over serious complications despite the lack of strong supporting data. This highlights the urgent need for robust, high-quality trials that distinguish between primary and incisional hernias [25], which also account for key patient subgroups such as those with obesity or prior surgical history. While technical outcomes and complication rates remain important, patient-reported outcomes, including chronic pain, foreign body sensation, recurrence, and quality of life, may represent the most meaningful measures of success in ventral hernia repair [38–40]. Placing greater emphasis on these outcomes can help guide future best practices that align with patient-centered care.

## Conclusion

Mesh placements believed to result in the best overall outcome varied by hernia defect size. For defects < 1 cm, preperitoneal and suture-only repairs were most commonly selected. For defects ≥ 1 to ≤ 4 cm, preperitoneal and retromuscular were equally favored, while for defects > 4 to 9 cm, retromuscular placement was selected by the majority of surgeons. Laparoscopic and robotic-assisted approaches were more commonly preferred with increasing defect size. Key patient characteristics influencing mesh placement decisions included hernia defect size, surgical history, and obesity. Despite clear trends in surgeon beliefs, there is very limited evidence supporting the mesh placements believed to yield the best outcomes, underscoring the need for further high-quality research.

## Data Availability

The data that support the findings of this study are available from the corresponding author upon reasonable request.
